# Association of Hypertension and Lipid Profile with Osteoporosis

**DOI:** 10.1155/2020/7075815

**Published:** 2020-07-12

**Authors:** Mohammed Al-Hariri, Bander Aldhafery

**Affiliations:** ^1^Department of Physiology, College of Medicine, Imam Abdulrahman Bin Faisal University, Dammam, Saudi Arabia; ^2^Department of Radiology, College of Medicine, Imam Abdulrahman Bin Faisal University, Dammam, Saudi Arabia

## Abstract

**Background:**

Hypertension (HTN) and osteoporosis (OP) are common diseases that adversely affect the health-related quality of life among the elderly. However, there is a scarcity of literature on the association between HTN and OP.

**Objective:**

The aims of this study were to investigate the association between HTN and antihypertensive drugs (AHT), with bone mineral density (BMD) *T*-scores, as well as to determine the status of bone quality in Saudi Arabia.

**Method:**

A retrospective study was conducted at King Fahd Hospital of the University, Khobar, Saudi Arabia, during 2016 to 2018. BMD was measured using dual-energy X-ray absorptiometry (DEXA). *T*-score values were used for the diagnosis of osteoporosis. HTN diagnosis and medications, laboratory, and radiology results were collected from the hospital record system.

**Results:**

Out of 1332 extracted profiles, 1103 (82.8%) were females. Based on the *T*-score, the majority of patients either had osteopenia (41.1%) or was osteoporotic (27.8%). The present study found that there is a significant increase in serum lipids and alkaline phosphatase (ALP) in the osteoporotic group when compared with normal and osteopenia groups. Furthermore, it was found that ALP and Ca levels were significant predictors for OP. Pearson's correlation test revealed a significant negative correlation between HTN and BMD *T*-score. However, the study reported a nonsignificant association between AHT and BMD *T*-score.

**Conclusion:**

We conclude that controlling both HTN and dyslipidemia might improve bone health. Every osteoporotic patient should be screened for dyslipidemia. Early detection and appropriate management for OP are highly recommended in Saudi Arabia, especially amongst the high-risk group.

## 1. Introduction

Osteoporosis (OP) is a systemic, chronic, metabolic bone disease characterized by impaired bone microstructure and decrease in bone mineral density (BMD) that predisposes a person to fractures [1].

In most of the cases, OP is a “silent disease” as it is not diagnosed until a fracture occurs. In addition, the routine spinal radiographs are not a useful method to detect OP until 30% of the bone is lost. According to the recommendation of the National OP Foundation, the diagnosis of OP is done by quantifying “BMD” by “dual-energy X-ray absorptiometry” (DEXA) of the hip, forearm, and spine with a *T*-score of ≤ −2.5 [2].

OP is considered as a major public health burden worldwide. Recent data estimated that over 200 million people in the world have OP, which is responsible for more than 8.9 million fractures/year [3].

An epidemiological report for Saudi Arabia in the year 2018 showed that 28.2% of men and 37.8% of women between 50 and 79 years of age were osteoporotic [4].

Hypertension (HTN) is the main risk factor for cardiovascular mortality, morbidity, and disability that adversely impacts both human capital and medical costs [5].

Globally, the prevalence of hypertension is increased with economic development and acceleration of population ageing, as well as modifications in lifestyle. According to reported data, HTN is a major cause of ischemic heart disease, stroke, and premature deaths [6].

In Saudi Arabia, the prevalence of HTN has been found 17.8% in males and 12.5% in females [7].

Many previous reports have shown similar biological basis in HTN and OP such as low level of nitric oxide, vitamin D, calcium (Ca), and vitamin K deficiency [8, 9]. To the best of our knowledge, no data have been reported on the association between HTN and OP in Saudi Arabia. Therefore, the aim of this study was to investigate the association between HTN and antihypertensive drugs (AHT), with BMD *T*-scores, in the hip and spine regions, as well as to determine the status of bone quality in Saudi Arabia using a retrospective analysis approach.

## 2. Methods

The current study was designed as a retrospective hospital-based analysis of patients who underwent BMD testing using the DEXA scan between the period of 2016 and 2018 at King Fahd Hospital of the University, Khobar, Saudi Arabia. The previous data that have been reported were from 2014 to 2016 [10].

The data were gathered using the hospital administrative software tool (PACS, Siemens AG, Erlangen, Germany).

DEXA “Hologic Discovery QDR Series, Marlborough, MA, USA, and software for Asians” is a standard way of assessing BMD and could provide data on *T*-scores, and it was interpreted according to the World Health Organization (WHO); *T*-scores represent the number of standard deviations below or above the average BMD, expressed in g/cm2; “based on the *T*-scores, patients are classified as follows: normal (>−1.0), with osteopenia (−1.0 to −2.5), and osteoporotic (≤−2.5),” and according to the WHO, an increase in the *T*-score indicates a worsening bone condition (i.e., the more negative the score, the worse the category) [11].

Data contained various kinds of medical and demographic information including age, gender, visits to the outpatient department, inpatient hospitalization, medications, diagnosis, and contact information of patients. Data also included several laboratory results that had been prescribed. This comprised cholesterol (Chol), triglycerides (TG), low-density lipoprotein (LDL), high-density lipoprotein (HDL), Ca, phosphorus (Ph), Vit D, and alkaline phosphatase (ALP). These results were extracted from the hospital's record system (QuadraMed).

All the laboratory values were determined according to the standard laboratory procedures.

The inclusion criteria comprised males and females between the age of 18 and 90 years who underwent DEXA scan for the lumbar spine and hip. Exclusion criteria were patients below 18 years and above 90 years as well as DEXA scan of other regions aside from the lumbar spine and hip joint.

This study was approved by the Institutional Review Board (IRB-2018-01-257) at the Deanship of Scientific Research, Imam Abdulrahman Bin Faisal University, Dammam.

### 2.1. Statistical Analysis

The analysis was done using IBM SPSS 26® software. Descriptive analysis, i.e., mean and standard deviation (SD), was used to report the results. One-way analysis of variance (ANOVA) was used to determine whether there were any statistically significant differences between the mean values of lipid and bone profiles between different bone quality categories. The chi-squared (*X*^2^) test was used for categorical variables. In addition, Pearson's bivariate correlation test was done to report the correlation between HTN and OP.

Lastly, a linear logistic regression analysis was conducted to predict the contribution of the independent variables to the *T*-score and to explore the relationships among them. Data with 95% confidence intervals (CI) and *P* value ≤ 0.05 were considered as statistically significant.

## 3. Results

Out of 1332 extracted profiles, 229 (17.2%) were males, and 1103 (82.8%) were females. Based on the *T*-score, the majority of patients either had osteopenia (41.1%) or was osteoporotic (27.8%) as tabulated in Table 1.

In Table 2, there was a significant increase in mean serum Chol (179 ± 47), LDL (106 ± 40), and ALP (91 ± 43) in the osteoporotic patient group when compared with normal (171 ± 39; *P* < 0.010, 98 ± 33; *P* < 0.04, and 80 ± 26; *P* < 0.0051) and osteopenia patient groups (173 ± 42; *P* < 0.038, 99 ± 35; *P* < 0.04, and 86 ± 34; *P* < 0.030), respectively. Additionally, mean serum ALP was significantly higher in the osteopenia patient group compared with normal (*P* < 0.013) as shown in Table 2.

When the *T*-score was predicted, it was found that ALP (beta < −003, *P* < 0.001), Ph (beta < −0.081, *P* < 0.041), and Ca (beta < −0.13, *P* < 0.018) levels were significant predictors.

The overall model fit was F(12) <2.6, *P* < 0.002, with an *R*^2^ of 0.05 (Table 3).

The two-way cross-tabulation and chi-squared test indicated that the majority of screened patients used combined AHT. The study also highlighted a nonsignificant association between the type of AHT with OP as shown in Table 4.

In Table 5, Pearson's bivariate correlation test revealed a significant correlation between HTN and BMD, and this relationship was negative (*r* = −0.28; *P* < 0.001).

## 4. Discussion

One of the most pertinent findings of this study was an abnormal lipid profile of the osteoporotic group as well as the prevalence of hospital-based osteopenia which appeared to be much higher among the patients. In addition, another prominent finding of the study was the loss of bone minerals in osteopenia and osteoporotic groups that was supported by the increased activity of ALP. Moreover, Pearson's bivariate correlation test revealed a significant negative correlation between HTN and BMD. Besides, Ca and ALP were found to be the independent predictors of osteopenia and OP.

In this study, most of the patients were prescribed with multiple therapies. Moreover, no association was found between AHT and OP. In addition, the incidence of low bone mass increased significantly with age and gender as females constituted a larger proportion. This may be attributed to the practice of recommending the DEXA scan for all females aged 65 and above as they were perceived to be more prone to having lower bone mineral density after menopause [12].

The link between HTN and OP has not received as much attention in terms of underlying pathophysiological mechanism, perhaps as both conditions are common in the elderly. Many reports suggested that both OP and HTN share some of the pathophysiological events, involving HTN is associated with high levels of the parathyroid hormone, which accelerate bone turnover, thus resulting in decreasing bone quality and bone mass [13]. Moreover, HTN is associated with a negative Ca balance and bone remodeling due to increased loss of Ca in the urine [14].

The present study revealed a significant negative correlation between HTN and BMD. These results coincide with several studies [15, 16]. These results suggest that a controlled HTN may have a beneficial role in bone quality in the elderly [16]. Moreover, the present results further highlight the health benefits of salt reduction both in terms of HTN and OP [17].

The results of the current study are inconsistent with another one conducted in Saudi Arabia in a different setting (smaller sample size and menopausal women) [18]. Regardless of the mechanisms involved, the present findings have several clinical contributions because HTN and OP are manageable in the elderly population.

Several studies have documented the negative impact of dyslipidemia and OP [19, 20]. Accumulating evidence documents that increased cellular cholesterol may inhibit osteoblast differentiation to prevent bone formation, whereas decreased cholesterol may prevent vascular calcification and bone loss by inhibiting osteoclast activity [21]. These arguments support our finding.

ALP is an isoenzyme, “a protein found in hypertrophic chondrocytes in the epiphyseal growth plate in calcifying matrix vesicles and mature osteoblasts.” It can provide valuable diagnostic information in some disorders such as metabolic bone disease which comprises approximately 50% of total circulating ALP [22].

ALP is considered to be a factor required for the mineralization and synthesis of a new bone, and it elevated as a result of increased osteoblastic activity and state of increased bone turnover [22].

Vit D has an important role in the maintenance of the healthy bone. This has been well reported in the literature. In the present work, the data showed no significant effect of Vit D on the *T*-score in females or males. Our findings are in agreement with a previous study conducted in Saudi Arabia [23]. It can be attributed to the achievement of the local recommended screening program for Vit D [10].

Detection of the early bone changes by ALP holds the key to the management of OP and as an effective indicator to reflect the efficiency of its management [24]. In the present study, ALP was found to be a good predictor and reflected the actual state of bone quality along with Ca.

## 5. Conclusion

We conclude that controlling both HTN and dyslipidemia might improve bone health.

Every osteoporotic patient should be screened for dyslipidemia. Early detection and appropriate management for OP are highly recommended in young Saudi population, especially the high-risk group, which includes women and patients with dyslipidemia, as well as those with high ALP, low sun exposure, and calcium intake.

## 6. Limitations

The present study was hospital-based and was conducted on a specific population. Therefore, the results cannot be generalized. A single bone biomarker was observed, and effect of confounding factors such as lifestyle and environmental factors on BMD was not studied.

## Figures and Tables

**Table 1:** Characteristics of the sample.

	Mean	SD
Cho (mg/dL)	174	43
TG (mg/dL)	120	61
LDL (mg/dL)	100	36
HDL (mg/dL)	52	14
ALP (IU/L)	86	35
Ph (mg/dL)	3.9	0.8
Vit D (ng/mL)	28	14
Ca (mg/dL)	9.1	0.6
Age	63	15

**Table 2:** Metabolic profile of the patients based on their mean *T*-score groups.

Mean ± SD (*P* value)	Normal	Osteopenia	Osteoporosis
	Mean ± SD (*P* value)	Mean ± SD (*P* value)	Mean ± SD (*P* value)
Cho (mg/dL)	171 ± 39	173 ± 42	179 ± 47 (0.010i) (0.038ii)
LDL (mg/dL)	98 ± 33	99 ± 35	106 ± 40 (0.04i) (0.04ii)
HDL (mg/dL)	52 ± 13	51 ± 14	51 ± 15
TG (mg/dL)	12761	124 ± 61	123 ± 58
ALP (IU/L)	80 ± 26	86 ± 34 (0.013ii)	91 ± 43 (<0.005i) (0.030ii)
Ph (mg/dL)	4 ± 0.8	4 ± 0.7	4 ± 0.7
Vit D (ng/mL)	27 ± 13	29 ± 15	28 ± 13
Ca (mg/dL)	9 ± 0.4	9 ± 0.6	9 ± 0.5

i: significantly higher than the normal group, ii: significantly higher than the osteopenia group, and iii: significantly higher than the osteoporosis group.

**Table 3:** Predictors of the measured *T*-score identified by multiple linear regression.

	*β* ^*∗*^ (95% CI)	*P* value
ALP	0.003 (0.001; 0.005)	0.001
Ca	−.130 (−0.24; −.020)	0.018
Ph	−.081 (−0.164; −0.003)	0.041
Vit D	.002 (−0.003; 0.006)	0.433
LDL	.006 (−0.003; 0.015)	0.168
TG	−7.478 (−0.002; 0.000)	0.916
Cho	−.004 (−0.012; 0.004)	0.314
HDL	.003 (−0.006; 0.012)	0.532
AHT	−.003 (−0.014; 0.035)	0.833

**Table 4:** Cross-tabulation of antihypertensive therapy to *T*-score categories.

Treatment	*T*-score			
	Normal	Osteopenia	Osteoporosis	Total
No	13	14	9	36
	36.1%	38.9%	25%	

ACE	22	16	17	55
	40.0%	29.1%	30.9%	

ARB	25	18	6	49
	51.0%	36.7%	12.2%	

CCB	24	47	25	96
	25.0%	49.0%	26%	

*β*-Blocker	18	22	14	54
	33.3%	40.7%	25.9%	
	1.8%	2.2%	1.4%	

Diuretics	11	6	8	25
	44.0%	24.0%	32%	

Combination	358	389	270	1017
	35.2%	38.2%	26.5%	

ACE: angiotensin-converting enzyme, ARB: angiotensin II receptor blockers, and CCB: calcium channel blockers.

**Table 5:** Correlation between HTN and BMD *T*-score.

Variable	BMD *T*-score *r* coefficient	*P* value
HTN	−0.28	<0.001

## Data Availability

The data used to support the findings of this study are included within the article.
